# The thienopyridine A-769662 and benzimidazole 991 inhibit human TASK-3 potassium channels in an AMPK-independent manner

**DOI:** 10.1016/j.bcp.2024.116562

**Published:** 2024-10-01

**Authors:** Esraa A. Said, Ryan W. Lewis, Mark L. Dallas, Chris Peers, Fiona A. Ross, Asier Unciti-Broceta, D. Grahame Hardie, A. Mark Evans

**Affiliations:** 1 Centre for Discovery Brain Sciences, Hugh Robson Building, University of Edinburgh, Edinburgh, EH8 9XD, UK; + Department of Pharmacology and Toxicology, Faculty of Pharmacy, Alexandria University; 2 School of Pharmacy, University of Reading, Reading RG6 6UB; 3 School of Life Sciences, University of Dundee, Dow Street, Dundee, DD1 4HN, UK; 4 Edinburgh Cancer Research, Cancer Research UK Scotland Centre, Institute of Genetics and Cancer, University of Edinburgh, Edinburgh, EH4 2XR, UK

**Keywords:** human TASK-3, AMPK, A-769662, 991, BI-9774, MK-8722, AICA-riboside, benzimidazole, 4-azabenzimidazole, thienopyridine

## Abstract

Heteromeric Tandem pore domain Acid Sensitive (TASK)-1/3 channels are critical to oxygen-sensing by carotid body type 1 cells, where hypoxia-induced inhibition of TASK-3 and/or TASK-1/3 potassium currents leads to voltage-gated calcium entry, exocytotic transmitter release and increases in carotid body afferent input responses that initiate corrective changes in breathing patterns. It was proposed that, in response to hypoxia, the AMP-activated protein kinase (AMPK) might directly phosphorylate and inhibit TASK channels, in particular TASK-3, but studies on rat type I cells questioned this view. However, sequence alignment identified a putative AMPK recognition motif in human (h) TASK-3, but not hTASK-1, with Ser^55^ representing a potential phosphorylation site. We therefore studied the effects of five different AMPK activators on recombinant hTASK-3 potassium channels expressed in human embryonic kidney (HEK)-293 cells. Two structurally unrelated AMPK activators, the thienopyridine A-769662 (100-500 µM) and the benzimidazole 991 (3-30 µM) inhibited hTASK-3 currents in a concentration-dependent manner, while the 4-azabenzimidazole MK-8722 (3-30 µM) partially inhibited hTASK-3 at concentrations above those required for maximal AMPK activation. By contrast, the 4-azabenzimidazole, BI-9774 (10-100 µM; a closely related analogue of MK8722) and the pro-drug AICA-riboside (1 mM; metabolised to ZMP, an AMP-mimetic) had no significant effect on hTASK-3 currents at concentrations sufficient to maximally activate AMPK. Importantly, A-769662 (300µM) also inhibited hTASK-3 channel currents in HEK-293 cells that stably over-expressed an AMPK-β1 subunit mutant (S108A) that renders AMPK insensitive to activators that bind to the Allosteric Drug and Metabolite site, such as A-769662. We therefore identify A-769662 and 991 as novel hTASK-3 channel inhibitors and provide conclusive evidence that AMPK does not regulate hTASK-3 channel currents.

## Graphical abstract

**Graphical Abstract:**
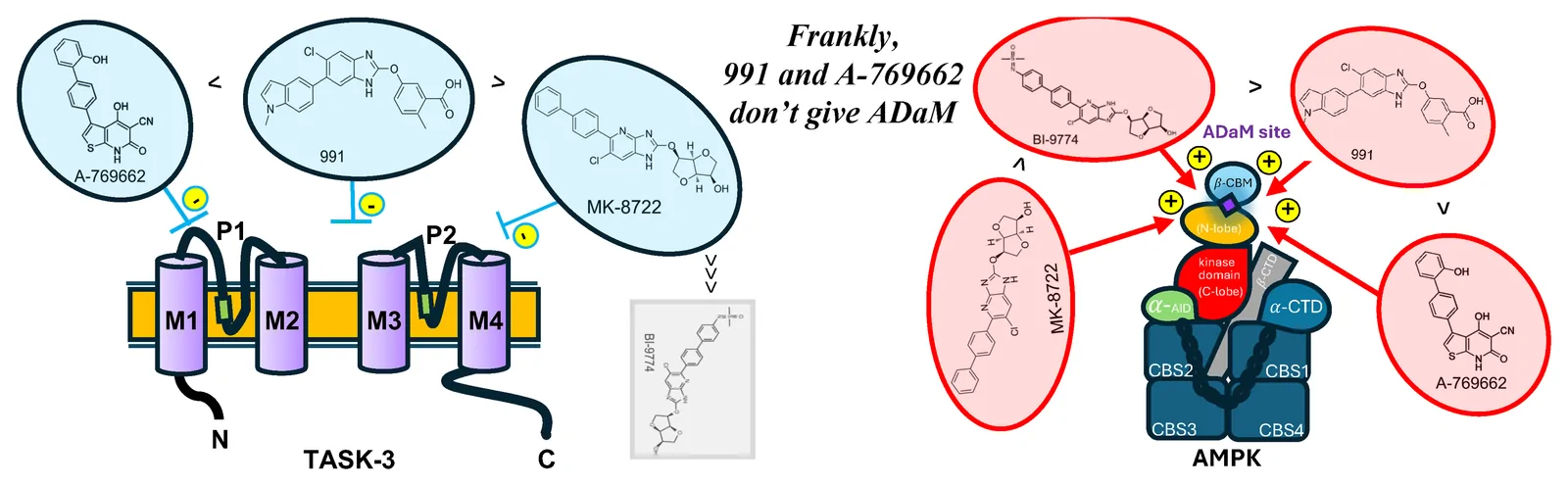

## Abbreviations

AMPK: AMP-activated protein kinase
TASK channels: Tandem (or Two) pore domain Acid Sensitive channels
HEK-293 cells: human embryonic kidney-293 cells
AICA-Riboside: Acadesine - 5-aminoimidazole-4-carboxaminde-1-beta-ribofuranoside
DMSO: Dimethylsulphoxide
PSS: physiological salt solution.

## 1 Introduction

We previously proposed that the AMP-activated protein kinase (AMPK) may mediate hypoxia-response coupling in type I cells of the carotid bodies, the primary peripheral arterial chemosensors, via direct phosphorylation and inhibition of heteromeric Tandem (or Two) pore domain Acid Sensitive (TASK) potassium channels and the large conductance voltage- and calcium-activated potassium channels (K_Ca_1.1) [1, 2], with studies on the former based primarily on the action of the AMPK activator AICA-riboside (5-AminoImidazole-4-Carboxamide-1-β-D-Ribofuranoside) which is metabolised by adenosine kinase to the AMP mimetic ZMP [2, 3]. However, we have since shown that while AMPK does indeed phosphorylate and inhibit K_Ca_1.1 [1], deletion of AMPK in mouse type I cells does not affect carotid body chemosensitivity [4, 5]. Furthermore, others have shown that TASK-3 homodimeric and TASK-1/3 heterodimeric channels comprise the dominant oxygen-sensitive potassium currents in rat carotid body type I cells [6, 7], and that two different AMPK activators, AICA-riboside and A-769662, are without effect on TASK-1/3 currents in rat carotid body type I cells [8], while AICA-riboside exhibits no effect on recombinant rat TASK-1 / TASK-3 homodimers or TASK-1/3 heterodimers [9].

Here, we have revisited the potential modulation by AMPK of human (h) TASK-3 channels, in which we identified a putative AMPK recognition motif. To this end we studied the action on hTASK-3 of the prodrug and AMP mimetic AICA-riboside and four direct AMPK activators, namely the thienopyridine A-769662 [10, 11], the benzimidazole 991 [10, 12], and the 4-azabenzimidazoles BI-9774 [13, 14] and MK-8722 [15, 16], all of which bind at the Allosteric Drug and Metabolite (ADaM) site between the α subunit kinase domain and the β subunit carbohydrate binding module. The 4-azabenzimidazoles MK-8722 [15, 17] and BI-9774 [14] are structurally similar and more potent AMPK activators than the benzimidazole 991, which is in turn 5-10 fold more potent an AMPK activator than the thienopyridine A-769662 [10]. Moreover, while MK-8722 [15, 16] and BI-9774 [13, 14] activate AMPK complexes containing either the β1 or β2 subunit isoforms [15, 16, 18], A-769662 [19, 20] and 991 (~10-fold) [10, 12] are β1-selective and reliant on AMPK-β1-S108 phosphorylation.

Here, we identify the thienopyridine A-769662 and the benzimidazole 991 as novel and potent hTASK-3 channel inhibitors, that act independent of AMPK. Moreover, we provide robust evidence that AMPK does not couple to recombinant hTASK-3 channels despite the presence of a potential AMPK recognition motif. These data also highlight the importance of using complementary approaches when using ligands to address the role of AMPK in cellular processes.

## 2 Materials and Methods

### 2.1 Generation of TASK-3 expressing cell lines

Human embryonic kidney (HEK)-293 cells were cultured in modified Eagle’s medium (MEM) with Earle’s salts and L-glutamine, supplemented with 10% (v/v) fetal calf serum (Globepharm, Esher, Surrey, UK), 1% (v/v) non-essential amino acids, 50 μg/ml gentamicin, 100 units/ml penicillin G, 100 μg/ml streptomycin and 0.25 μg/ml amphotericin in a humidified atmosphere of air/CO_2_ (19:1) at 37°C. All cell culture reagents were purchased from Gibco™ (Thermo Fisher, Paisley Scientific, UK) unless otherwise stated.

HEK-293 cells stably expressing FLAG-tagged AMPK-β1 with a S108A mutation that renders AMPK insensitive to activation by drugs acting via the ADaM site, such as A-769662 [19]; in these cells the FLAG-tagged β1 subunit effectively replaces the endogenous β1 and β2 subunits.

Full length cDNAs encoding human TASK-3 (hTASK-3) channels, a kind gift from Dr. S. A. N. Goldstein (Department of Pediatrics and Institute for Molecular Pediatric Sciences, Pritzker School of Medicine, University of Chicago), were originally subcloned into the mammalian expression vector pMAX(+) via Xba I/Xma I restriction site combinations.To generate HEK-293 cell lines stably expressing wild-type hTASK-3, cells were transfected with pMAX/hTASK-3 constructs, using the PolyFect transfection reagent (Qiagen, Hybaid Ltd, Teddington, UK) according to manufacturer’s instructions. Stable HEK-293 cell lines were achieved by antibiotic selection with G-418 (1mg/ml, Geneticin™, ThermoFisher Scientific Company, Paisley, UK) added to the medium 3 days after transfection. Selection was applied for 4 weeks (media changed every 4-5 days), after which time individual colonies were picked and seeded in T25 flasks and allowed to reach confluence. They were then transferred to T75 flasks for further culture and electrophysiological screening. Cells were harvested from culture flasks by trypsinization and plated onto coverslips 24-48h before use in electrophysiological studies.

Transfection of hTASK-3 channels was considered successful if the currents elicited by the whole-cell voltage ramp protocol (see section 2.3 below and Figures 2-6) were: a) significantly (> 4-fold) larger than untransfected HEK-293 cell K^+^ currents; b) reliably described by the Goldman Hodgkin and Katz (GHK) equation; c) activated by pH 8.4 and inhibited by pH 6.4 (and the pH-sensitive currents demonstrated GHK rectification). In addition, the presence of hTASK-3 channel currents was confirmed by inhibition with 100µM ruthenium red, as previously described [21]. Transient transfection of HEK-293 cells that stably over-expressed a FLAG-tagged AMPK-β1 S108A mutant [19] with recombinant hTASK-3 channels was verified in a similar manner.

### 2.2 Cell transfection

hTASK-3 was overexpressed by transient transfection transient transfection (Invitrogen™ Lipofectamine^®^ 2000 Transfection Reagent, ThermoFisher Scientific Company, Paisley, UK) into a HEK-293 cell line stably over-expressing AMPK-β-1S108A mutant subunit, that were seeded on Poly-D-Lysine-coated coverslips (50µg/ml, Sigma-Aldrich Company Ltd., Dorset, UK). At 48h post-transfection, cells were washed with bath solution and used for electrophysiology.

### 2.3 Electrophysiology

Whole-cell patch-clamp recordings were acquired from either HEK-293 cells stably expressing hTASK-3, or HEK-293 cells stably expressing the AMPK-β1 S108A mutant that were transiently expressed wild-type hTASK-3. Coverslips with attached cells were transferred to a continuously perfused recording chamber (perfusion rate 3-5 ml/min, volume ca 200μl) mounted on the stage of an inverted microscope (Zeiss, Axioscope). Cells were perfused with a solution containing (in mM): 135 NaCl, 5 KCl, 1.2 MgCl_2_, 5 HEPES, 2.5 CaCl_2_, 10 D-glucose (pH 7.4 with KOH). Patch electrodes (resistance 4-7MΩ) were filled with intracellular solution consisting of (in mM): 10 NaCl, 117 KCl, 2 MgCl_2_, 11 HEPES, 11 EGTA, 1 CaCl_2_, 2 Na_2_ATP (pH 7.2 with KOH).

Outward K^+^ currents were recorded at 37°C (unless otherwise stated) using standard ramp protocols; cells were voltage-clamped at a holding potential of -70mV, then stepped to -100mV and a voltage ramp (500ms duration) immediately applied from -100 mV to +60mV. Data were acquired and digitized via a Digidata 1322A in combination with an Axopatch 200B amplifier and Clampex 10 software (Molecular Devices, Foster City, CA). Currents were sampled at 2kHz and low-pass filtered at 1kHz. Offline data analysis was conducted using Clampfit 10 software (Molecular Devices, Foster City, CA).

AMPK agonists tested here were applied extracellularly once hTASK-3 current magnitude stabilised after entering the whole-cell configuration; usually 3-4 successive and consistent measures, with I_0_ taken as the average of the final 2 measures prior to drug application. The mean ± SEM for I/I_0_ x 100 was measured after current equilibration, the time for which varied between cells, AMPK agonists and concentrations applied. For control experiments (physiological salt solution (PSS) or 0.1% Dimethylsulphoxide (DMSO)) current magnitude was measured at time points (10min) equivalent to the longest period required for hTASK-3 current inhibition by the AMPK agonists tested here.

### 2.4 Drugs and chemicals

All chemicals were obtained from Sigma-Aldrich (Poole, UK) except MK-8722 (synthesized using published methods[18] by Dr Natalia Shpiro, University of Dundee,) and BI-9774 (A kind gift from David Carling, Imperial College London). Drugs were applied to cells by direct bath application.

### 2.5 Statistical analysis

Data analysis was carried out using Graphpad Prism 10.0. Statistical analysis was completed using the paired Student’s t-test, unpaired Student’s t-test, One-way ANOVA followed by Holm-Sidak’s post-hoc test. Differences were considered significant when P<0.05. All values stated as mean ± SEM.

## 3 Results

### 3.1 Recombinant hTASK-3 channels have an AMPK recognition motif

**Figure 1:**
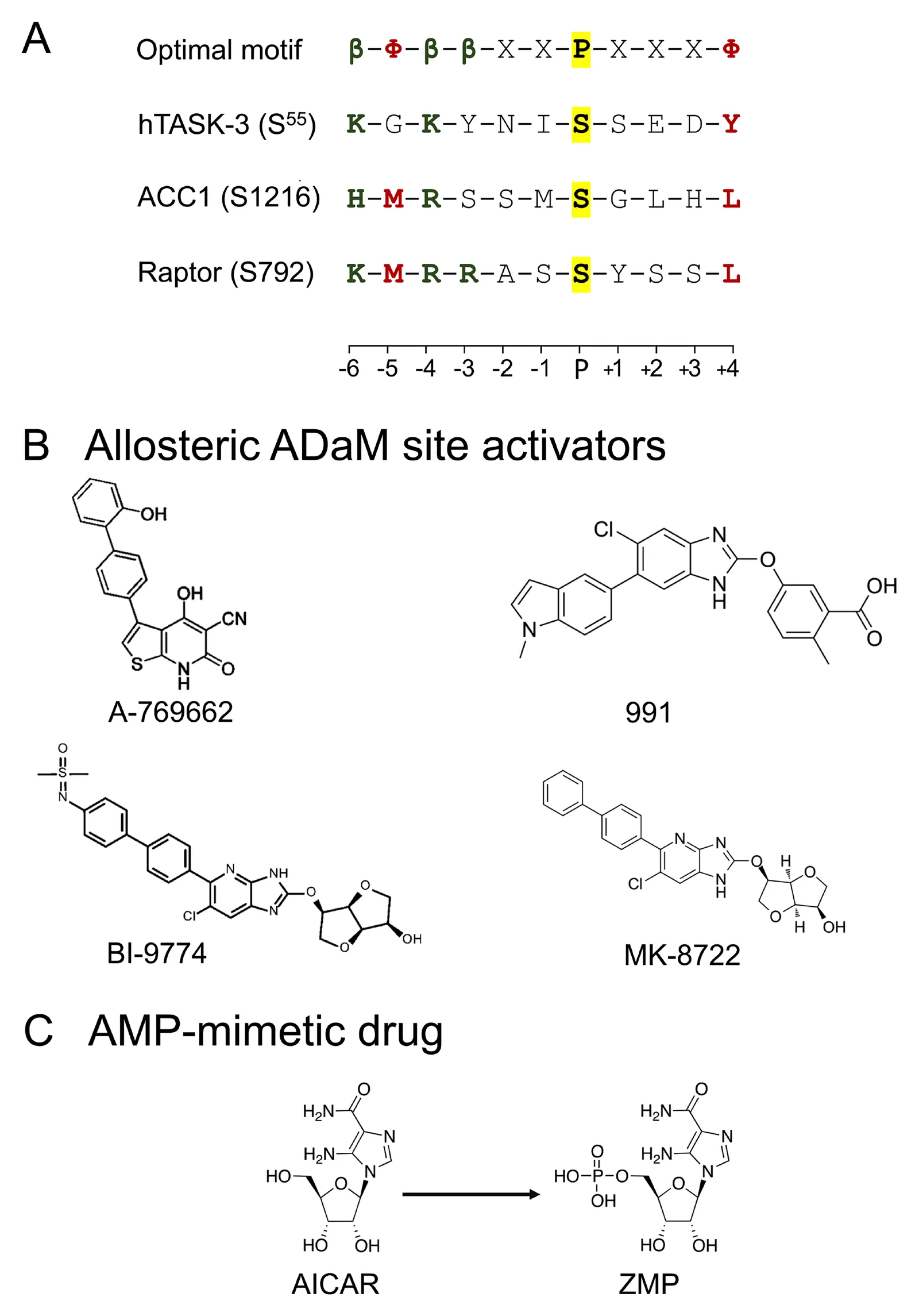
A putative AMPK recognition motif in human TASK-3 channels and molecular structures of the AMPK agonists A-769662, 991, BI-9774, MK-8722 and AICA-riboside. A, Alignment of putative recognition motifs for the AMPK-activated protein kinase (AMPK). Upper sequence is an idealised motif. The sequences below are the putative AMPK recognition motif for human (h)TASK-3, and known recognition motifs for acetyl-CoA carboxylase (ACC1) and Raptor. P represents phosphorylation sites, ϕ hydrophobic residue and β basic residues that are likely involved in AMPK recognition. B, Chemical structures of the four compounds tested here that activate AMPK by binding to the allosteric drug and metabolite (ADaM) site, namely A-769662, 991, BI-9774 and MK-8722. C, Chemical structure of the pro-drug AICA-riboside and its metabolite and AMP-mimetic ZMP.

We examined the amino acid sequence of human (h) TASK-1 and hTASK-3 for a motif comparable to those found in recognised AMPK substrates, and identified a putative AMPK recognition motif in hTASK-3, but not hTASK-1. Figure 1A illustrates an optimal AMPK recognition motif, with idealised hydrophobic (Φ) and basic (β) residues surrounding a candidate serine residue (putative phosphorylation site). Sequence alignment indicated that Ser^55^ in hTASK-3 represented a possible candidate site for AMPK-directed phosphorylation (Fig. 1A), although this is not a perfect fit to the AMPK recognition motif because it lacks a bulky hydrophobic residue at the P -5 position, while the tyrosine at the P +4 position may also be sub-optimal [22, 23]. However, we were unable to recover sufficient channel protein to determine whether or not ^32^P incorporation from radiolabelled ATP was induced by purified AMPK as we have done successfully for other ion channels regulated by AMPK, such as K_V_1.5, K_V_2.1 and K_Ca_1.1 [24–27].

### 3.2 A-769662 and 991 inhibit hTASK-3 currents in a concentration-dependent manner

**Figure 2:**
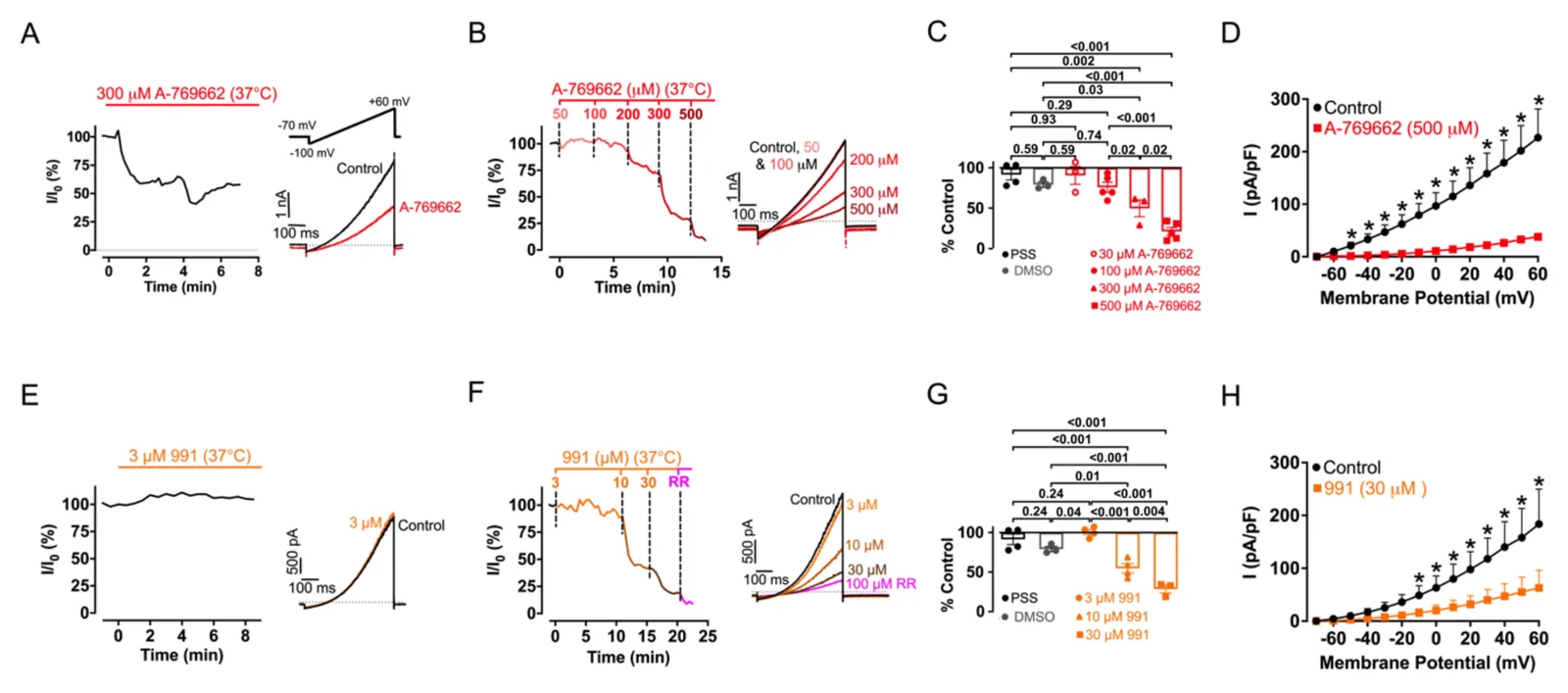
A-769662 and 991 inhibit human TASK-3 potassium currents in a concentration-dependent manner. A, Normalized human (h)TASK-3 current at +60 mV versus time before and after application of 300μM A-769662 (left), and instantaneous current-voltage relationships (right) recorded during voltage ramps from -100 mV to +60 mV (holding potential -70 mV, 37° C) for hTASK-3 currents stably expressed in HEK-293 cells. B, as in A but shows concentration-dependent inhibition of hTASK-3 currents by A-769662 (50-500μM). C, Bar graphs and scatter plots show normalized currents (% control (I_0_)) carried by hTASK-3 stably expressed in HEK-293 cells in the absence (physiological salt solution, PSS) and presence of 0.1% DMSO and after extracellular application of A-769662 (30, 100, 300, and 500 µM). Results are expressed as mean ± SEM (n = 4-5). Statistical analysis was by one-way ANOVA followed by Holm-Sidak’s post-hoc test. D, Instantaneous current density-voltage relationships for hTASK-3 currents recorded during voltage ramps before and after extracellular application of 500 μM A-769662. Results are expressed as mean ± SEM (n = 3-4). Statistical analysis was carried out by paired t-test. * = P < 0.05. E as in (A) but for 3µM 991. F as in (B) but shows concentrsation-dependent inhibition of hTASK-3 by 991 (3-30 µM), followed by bath application of 100 µM Ruthenium Red (RR). G, as in (C) but after after extracellular application of 991 (3, 10 and 30 µM). H, as in (D) but before and after extracellular application of 30 μM 991.

In marked contrast to the findings of others who identified no effect of 500 µM A-769662 on TASK-1/3 channel activity in rat carotid body glomus cells [8], we found that the thienopyridine A-769662 virtually abolished hTASK-3 currents at similar concentrations. In this respect, it is notable that when first applied A-769662 induced a transient increase in peak outward current at +60 mV that measured (mean ± SEM, I/I_0_ x 100) 119.84 ± 6.54 % of control at 3 µM (n = 3, P = 0.045) and 110.91 ± 3.14 % at 500 µM (n =3, P = 0.04) prior to the onset of current inhibition (Fig. 2A). Subsequently, hTASK-3 current inhibition was observed at concentrations >100 µM, which occured in a concentration-dependent manner and reached a maxmimum within 5 min (Fig. 2A-D). This resulted in significant reductions in peak current measured at +60 mV relative to the point of application (time zero) for 300 µM A-769662 (mean ± SEM for I/I_0_ x 100: 49.76 ± 10.36 %; n = 3; 1.67 ± 0.17 min to equilibration; P = 0.002 versus PSS; P = 0.03 versus 0.1 % DMSO) and 500 µM A-769662 (mean ± SEM: 21.20 ± 4.83 %, n = 5, 3.10 ± 0.70 min to equilibration; P <0.001 versus PSS and 0.1 % DMSO) when compared to measures from the control groups at 10 min (mean ± SEM for I/I_0_ x 100: PSS, .91.48 ± 6.68 % at 10 min, n = 4; 0.1 % DMSO, 79.17 ± 3.28 % at 10 min, n = 3).

Moreover, hTASK-3 currents were also inhibited in a concentration-dependent manner by the benzimidazole 991 (10-30 µM; Fig. 2E-H), which is structurally unrelated to A-769662. Significant reductions in peak current were measured at +60 mV following bath application of 10 µM (mean ± SEM for I/I_0_ x 100: 54.75 ± 5.82 %; n =4; P < 0.001 versus PSS, P = 0.01 versus DMSO; 5.75 ± 1.16 min to equilibration) and 30 µM 991 (mean ± SEM, 28.53 ± 4.96; n = 3; P <0.001; 4.83 ± 0.73 min to equilibration) when compared to controls at 10 min, with very small residual current remaining after application of 30 µM 991 (Fig. 2. F) that was identified by application of the TASK-3 inhibitor ruthenium red (100 µM) [21].

In this respect it is notable that, unlike A-769662, when first applied 991 did not induce a transient increase in hTASK-3 currents prior to the onset of current inhibition; see discussion for further details.

### 3.3 The pan-AMPK activators MK-8722 and BI-9774 are less effective inhibitors of recombinant hTASK-3 channels

In marked contrast to the effects of A-769662 and 991, two 4-azabenzimidazoles that are pan-β AMPK activators, namely BI-9774 and MK-8722, were found to be less effective inhibitors of hTASK-3.

**Figure 3:**
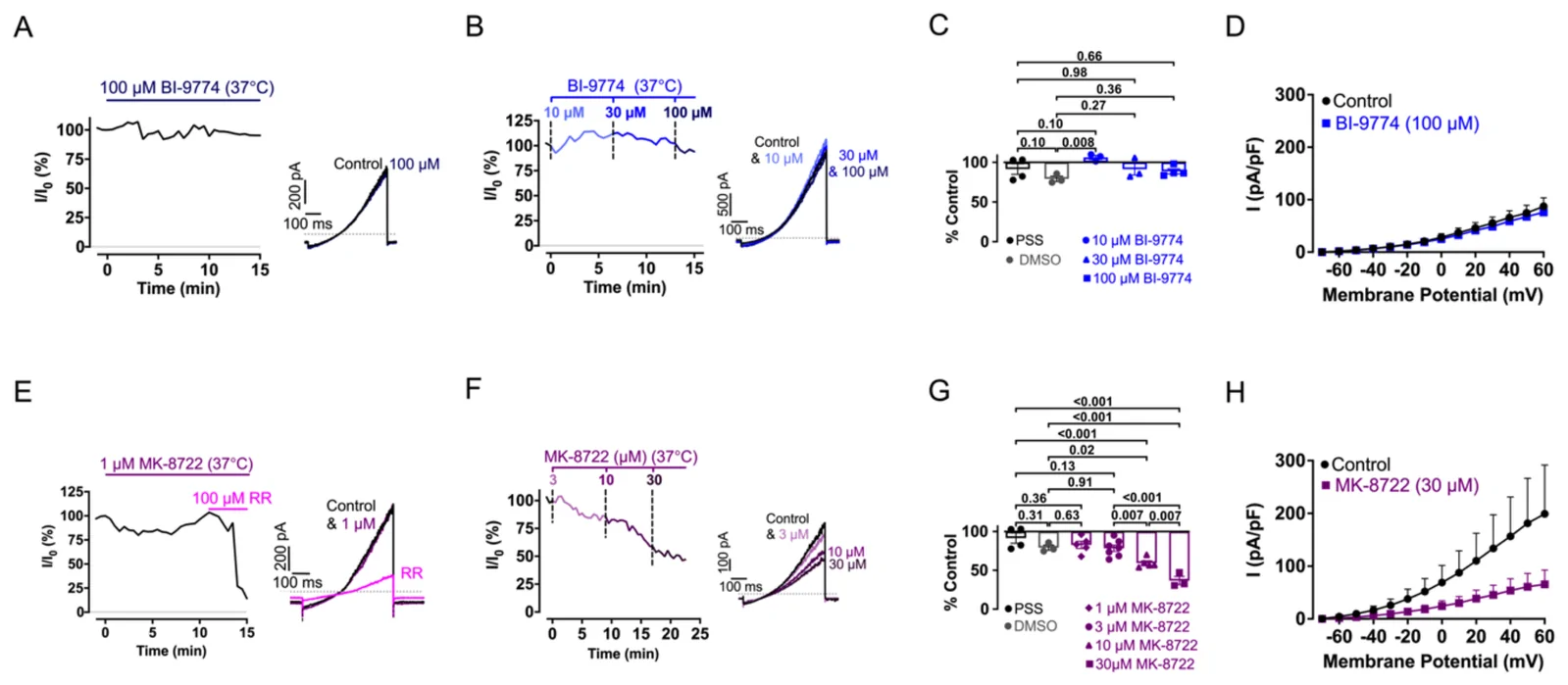
Effect of the pan-AMPK activators BI-9774 and MK-8722 on human TASK-3 potassium channel currents. A, Normalized human (h)TASK-3 current at +60 mV versus time before and after application of 100 μM BI-9774 (left), and instantaneous current-voltage relationships (right) recorded during voltage ramps from -100 mV to +60 mV (holding potential -70 mV, 37° C) for hTASK-3 currents stably expressed in HEK-293 cells. B, as in (A) but shows the effect on hTASK-3 currents of cumulative increases in BI-9774 concentration (10-100μM). C, Bar graphs and scatter plots show normalized currents (% control (I_0_)) carried by hTASK-3 channels stably expressed in HEK-293 cells in the absence (physiological salt solution, PSS) and presence of 0.1% DMSO and after extracellular application of BI-9774 (10, 30, and 100 µM). Results are expressed as mean ± SEM (n = 3-4). Statistical analysis was by one-way ANOVA followed by Holm-Sidak’s post-hoc test. D, Instantaneous current density-voltage relationships for hTASK-3 currents recorded during voltage ramps before and after extracellular application of 100 μM BI-9774. Results are expressed as mean ± SEM (n = 4). Statistical analysis was carried out by paired t-test. * = P < 0.05. E as in (A) but for 1µM MK-8722. F as in (B) but shows concentration-dependent inhibition of hTASK-3 by MK-8722 (3-30 µM). G, as in (C) but after after extracellular application of MK-8722 (1, 3, 10 and 30 µM). H, as in (D) but before and after extracellular application of 30 μM MK-8722.

BI-9774 is a more potent AMPK activator than 991, yielding maximal activation at 10 µM [13, 14]. Despite this fact, BI-9774 had little or no effect on hTASK-3 potassium currents when bath applied at 10-100 µM (Fig. 3A-D). After 5 min hTASK-3 potassium currents measured (mean ± SEM for I/I0 × 100) 106.20 ± 2.50 % for 10 µM B-I9774 (n=3), 91.33 ± 7.36 % for 30 µM BI-9774 (n=3) and 88.59 ± 3.24 % (n=4) for 100 µM BI-9774 relative to the point of application, which was not significantly different from controls. Moreover, little or no effect was observed in one cell exposed to 100 µM BI-9774 for 20 min (Fig. 3A).

At concentrations (1 µM) sufficient to induce maximal AMPK activation [15, 16] [28], MK-8722 also failed to significantly inhibit hTASK-3 currents when compared to control (Fig. 3E and G). Time-dependent rundown of hTASK-3 currents was evident in some cells but neither the spread nor the average of the data points was significantly different from controls (Fig. 3G). That said, at higher concentrations MK-8722 (3-30µM) partially inhibited hTASK-3 currents and did so in a concentration-dependent manner (Fig. 3E-H). Reductions in hTASK-3 currents did not reach significance for 3µM MK-8722. However, significant reductions in peak current were measured at +60 mV following bath application of 10 µM (mean ± SEM for I/I_0_ × 100: 59.35 ± 2.81 %, n = 5, 7.60 ± 0.83 min to equilibration; P = 0.001 versus PSS; P = 0.02 versus 0.1 % DMSO) and 30µM MK-8722 (mean ± SEM for I/I_0_ × 100: 36.70 ± 5.27 %, n = 3, 5.50 ± 0.29 min to equilibration; P <0.001 versus PSS and 0.1 % DMSO) when compared to control groups at 10 min. It is important to note, however, that the structure of MK-8722 is very similar to that of BI-9774 (Fig. 1B), and at concentrations ≥3 µM MK-8722 likely inhibits mitochondrial oxidative phosphorylation [28]; see discussion for further details.

**Figure 4:**
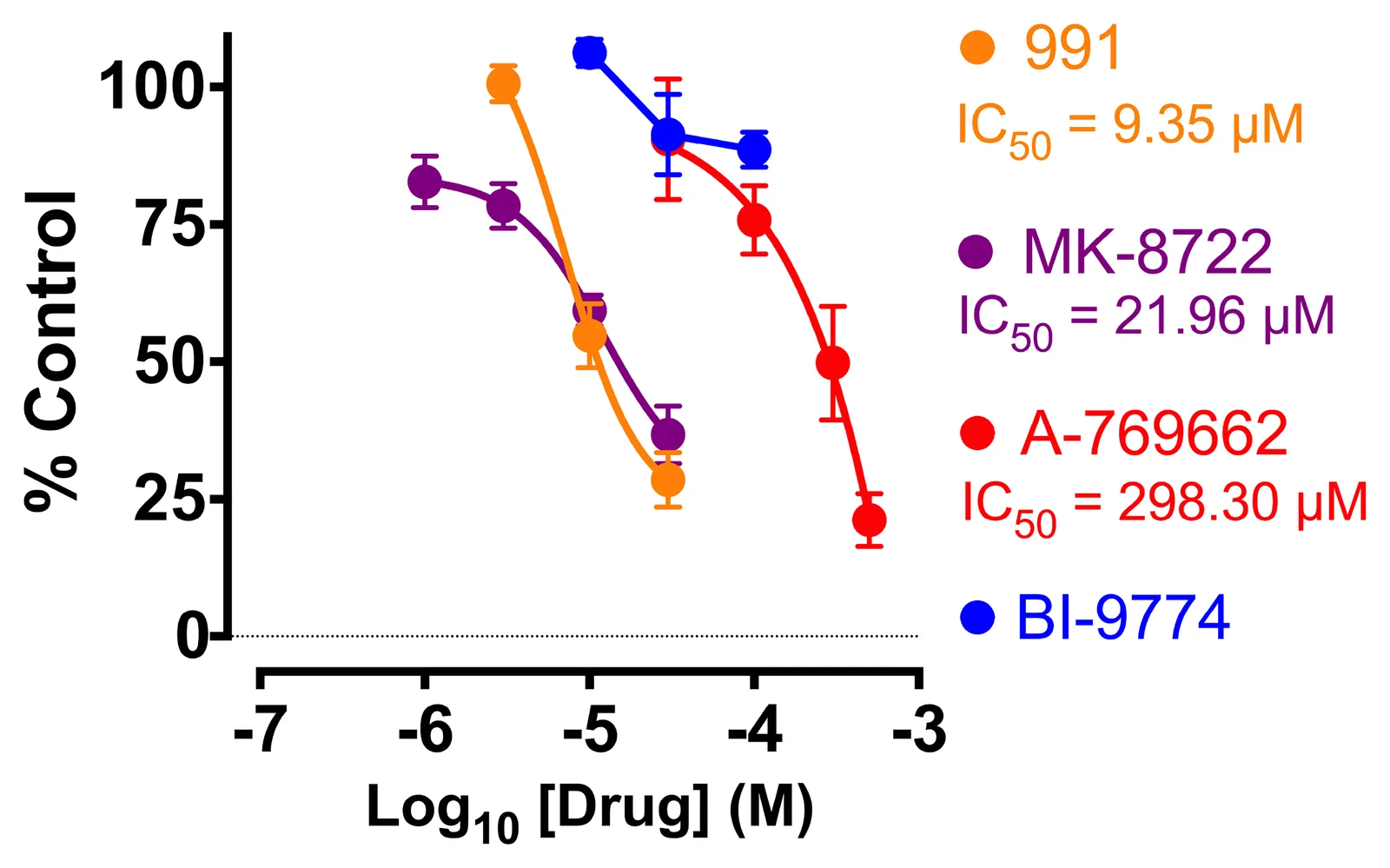
Concentration-inhibition curves for the effect on human TASK-3 potassium current of 991, A-769662, MK-8722 and BI-9774. Concentration-inhibition curves for the inhibition of human (h)TASK-3 potassium currents by 991, A-769662, MK-8722 and BI-9774, and the IC_50_ values obtained by interpolation. Curves for 991, A-769662 and MK-8722 were obtained by best fit to a hill equation.

The aforementioned findings are significant because the IC_50_ for hTASK-3 channel inhibition was 9.4 µM for 991 > 22 µM for MK-8722 > 298.3 µM for A-769662 (Fig. 4), yielding a rank order of potency that is different from that obtained for direct activation of AMPK by binding of these inhibitors to the ADaM site, which reads MK-8722 > BI-9774 > 991 > A-769662; see discussion for further details.

The Hill coefficient from the best fit to the concentration-inhibition curve for 991 was - 1.93, suggesting positive cooperativity; although this finding should be treated with caution due to the small number of data points fitted. By contrast, the Hill coefficients for A-769662 and MK-8722 were closer to 1.

### 3.4 The pro-drug AICA-Riboside has no effect on hTASK-3 currents

**Figure 5:**
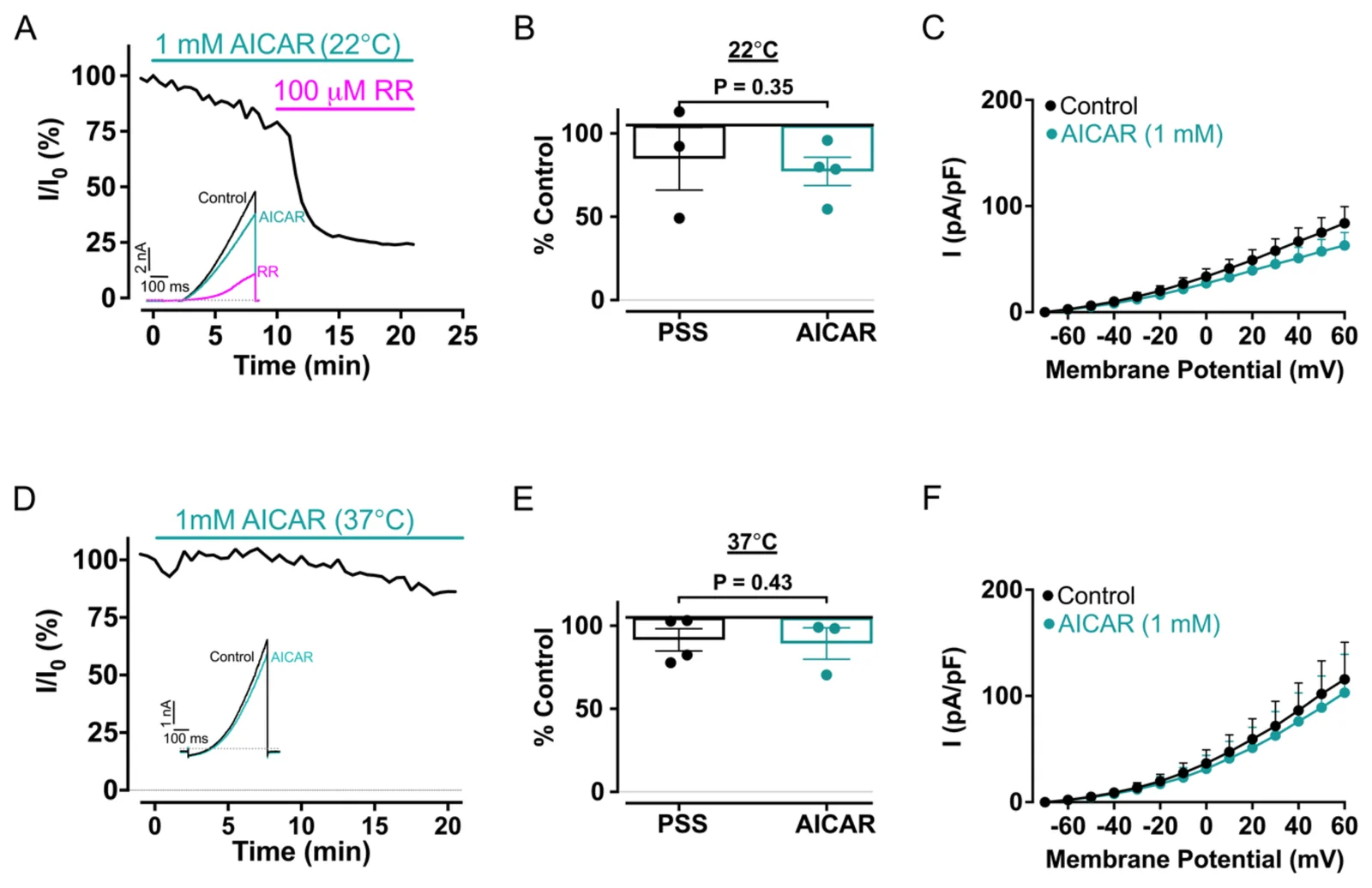
The pro-drug and AMP-mimetic AICA-riboside has no effect on potassium currents carried by human TASK-3 channels stably expressed in HEK-293 cells. A, Time course shows the effect of extracellular application of 1mM AICA-riboside and then 100μM Ruthenium Red (RR) on normalized human (h)TASK-3 current magnitude (I/I_0_) at the peak (+60 mV) of instantaneous current-voltage relationships recorded during voltage ramps from -100 mV to +60 mV (holding potential -70 mV, 22° C); Insets, show example records of the instatneous current-voltage relationship for hTASK-3 currents before and after extracellular application of 1mM AICA-riboside alone or in combination with 100 μM RR. B, Bar graphs and scatter plots illustrate normalized hTASK-3 currents (% of control (I_0_)) stably expressed in HEK-293 cells following application of 1mM AICA-riboside (20 min) at 22° C. Results are expressed as mean ± SEM, n =3-4. Statistical analysis was carried out by unpaired t-test. PSS, physiological salt solution C, Instantaneous current density-voltage relationships recorded during voltage ramps for hTASK-3 current before and after extracellular application of 1mM AICA-riboside at 22° C. Results are expressed as mean ± SEM (n = 4). Statistical analysis was carried out by paired t-test. D, E and F as in (A), (B) and (C) but for the effect of 1 mM AICA-riboside at 37° C.

Consistent with the lack of effect of BI-9774, the pro-drug AICA-riboside (1 mM; which yields the AMP mimetic ZMP) had no effect on hTASK-3 currents at 22° C (Fig. 5A-C) at time points twice as long as the time required for maximal effect on ion channels studied previously [27]. Briefly, hTASK-3 currents measured (mean ± SEM for I/I0 × 100) 77.19 % ± 8.52 (n = 4) at 20 min, which was not significantly different from PSS alone after 10 min (84.77 ± 18.82 %; n = 3; P=0.35); and this was confirmed by extracellular application of ruthenium red (100 µM). Given that AICA-riboside is a pro-drug that undergoes conversion by cellular uptake and phosphorylation by adenosine kinase (Fig. 1C) [29], we repeated the same experiment at 37°C (Fig. 5D-F) and confirmed that extracellular application of 1 mM AICA-riboside had little or no inhibitory effect on hTASK-3 channel currents at 10 min, 17 min and 20 min, with no significant difference measured at 10 min (mean ± SEM for I/I_0_ × 100: 89.26 ± 9.44%; n = 3; P = 0.43) when compared to controls (mean ± SEM for I/I_0_ × 100: PSS, 91.48 ± 6.68%; n = 4).

### 3.5 A-769662 inhibits hTASK-3 currents in HEK-293 cells stably overexpressing the AMPK-β1 S108A mutant

**Figure 6:**
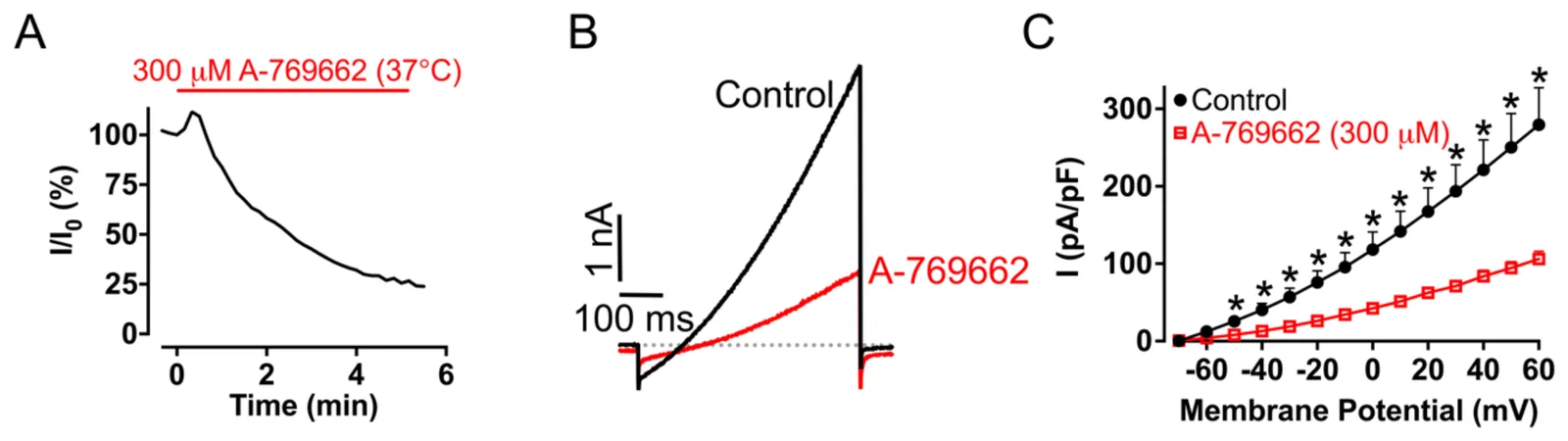
The effect of the AMPK-β1 S108A mutant on inhibition by A-769662 of human TASK-3 potassium channel currents. A, Time course for the effect on normalized human (h)TASK-3 current magnitude (I/I_0_, where I_0_ = average current at point of application) at the peak (+60mV) of the instantaneous current-voltage relationships recorded during voltage ramps from - 100mV to +60mV (holding potential -70mV, 37° C) before and during extracellular application of 300 μM A-769662 (red line). B, Instantaneous current density-voltage relationships recorded during voltage ramps from -100mV to +60mV (holding potential -70mV, 37° C) for hTASK-3 currents transiently over-expressed in HEK-293 cells stably over-expressing an AMPK-β1 S108A mutant, before and 5 min after extracellular application of 300μM A-769662. C, Instantaneous current density-voltage relationships for hTASK-3 recorded during voltage ramps before and ~5min after extracellular application of 300μM A-769662 at 37° C. Results are expressed as mean ± SEM (n = 6). Statistical analysis was carried out by paired t-test. *P < 0.05.

Given that neither BI-9774 nor AICA-riboside had a significant effect on hTASK-3 current magnitude, we concluded that the observed inhibition of hTASK-3 currents with the AMPK-β1 selective A-769662 and 991 was mediated in an AMPK-independent manner. To confirm this, we selected as our lead agonist A-769662. A-769662 binds to the ADaM site on AMPK (as do 991, MK-8722 and BI-9774) in the cleft between the N-lobe of the kinase domain on the α subunit and the Carbohydrate-Binding Module on the β subunit (β-CBM) [10, 11], phosphorylation of which at Ser^108^ by upstream kinases stabilises the ADaM site by facilitating interaction between two lysine residues in the kinase domain N-lobe. An S108A mutation destabilises the ADaM site and essentially abolishes the effects of ADaM site activators such as A-769662 [10, 19]. We therefore tested the effect of 300 µM A-769662 (~IC_50_) on hTASK-3 channels transiently expressed in HEK-293 cells that stably over-expressed a FLAG-tagged AMPK-β1 S108A mutant; by competing for binding to the α and γ subunits AMPK-β1 S108A essentially replaces the endogenous β1 and β2 subunits [19]. In cells stably over-expressing AMPK-β1 S108A, extracellular application of 300 µM A-769662 induced small but significant increases in outward current (Fig.6A, mean ± SEM for I/I_0_ × 100: 111.34 ± 4.01 %; n = 6; P = 0.02) that were not significantly different from wild type (mean ± SEM for I/I_0_ × 100: 110.91 ± 3.14 %; n = 3). This was followed by, consistent with a direct action on hTASK-3, robust reductions in hTASK-3 currents (Fig. 6, mean ± SEM for I/I_0_ × 100: 44.01 ± 5.86 %; n = 6) which were not significantly different from that seen for cells expressing wild type AMPK-β1 (mean ± SEM for I/I_0_ × 100: 49.76 ± 10.36 %; n = 3; P = 0.31).

## 4 Discussion

**Figure 7:**
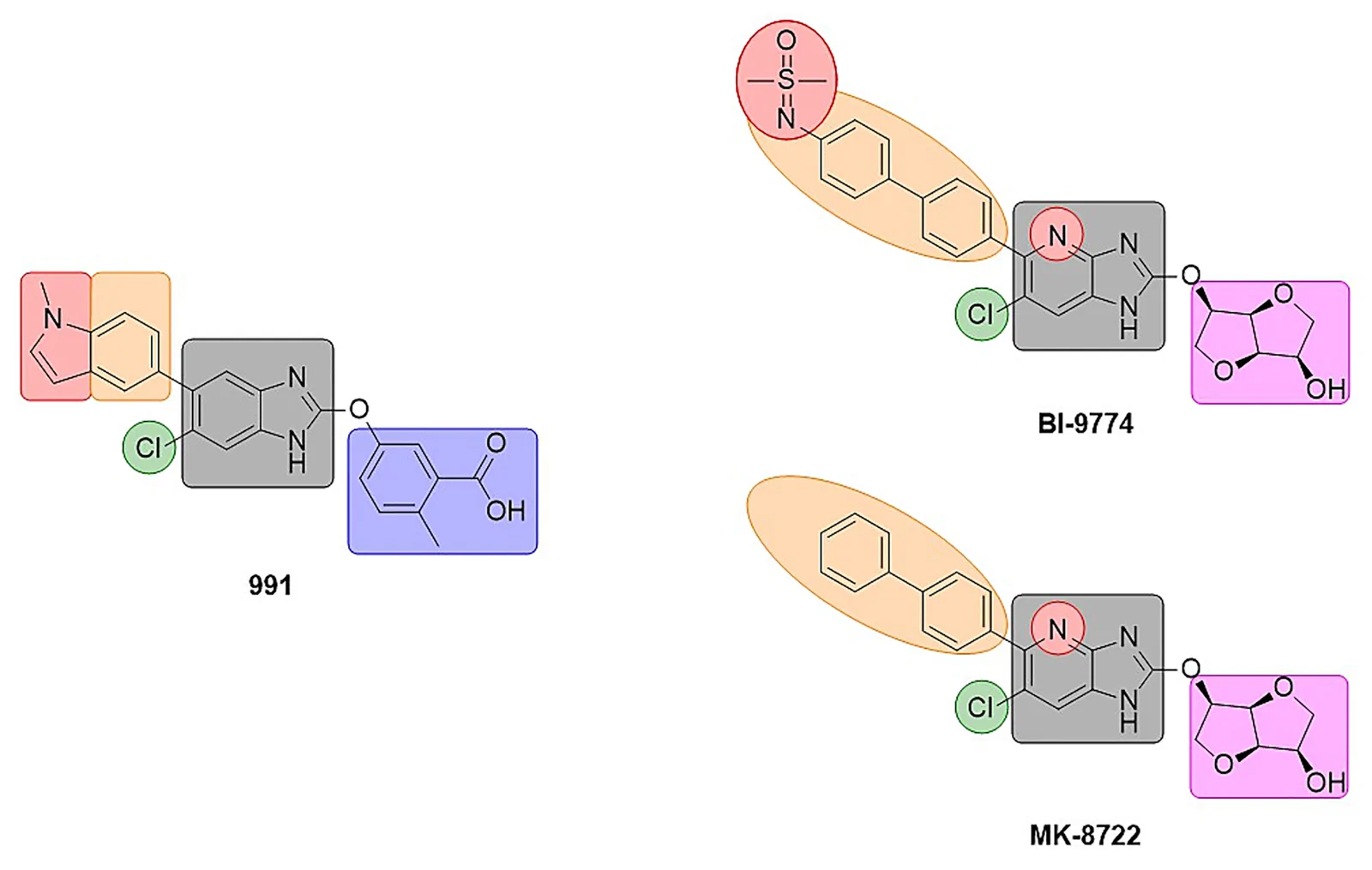
Analysis of the structural similarities between compounds 991, BI-9774 and MK-8722. The central benzimidazole core and the chloro substituent, shared by the three compounds, are highlighted in grey and green, respectively. BI-9774 and MK-8722 contain an endocyclic nitrogen in their core (highlighted in red), but 991 does not. Both BI-9774 and MK-8722 have a dianhydro-D-mannitol moiety attached to the C2 position of the central ring (highlighted in purple), whereas 991 has an arylcarboxylate group connected through an ether linkage at that position (highlighted in blue). The only structural difference between BI-9774 and MK-8722 is the red-highlighted imino-dimethyl-lambda6-sulfanone group, which is only present at the para position of the orange-highlighted biphenyl ring of BI-9774.

The present study strongly suggests that hTASK-3 potassium currents are strongly inhibited in a concentration-dependent, voltage-independent and AMPK-independent manner by two structurally unrelated compounds (Fig. 7), namely the thienopyridine A-769662 (100-500 µM) and the benzimidazole 991 (10-30 µM), each of which selectively activates AMPK-β1 containing isoforms by binding to the allosteric drug activation and metabolite (ADaM) site. In each case, the effective concentration range partially spanned that across which A-769662 and 991 activate AMPK [10, 12].

In stark contrast, we found that hTASK-3 currents remained unaffected in the presence of the 4-azabenzimidazole pan AMPK-β activator BI-9774 (Fig. 1), even when applied at concentrations (100 µM) an order of magnitude higher than the concentration (10 µM) required for maximal AMPK activation through binding to the ADaM site [14]. In this respect, it is notable that while BI-9774 has a similar benzimidazole core to that of 991 and chloro groups attached in equivalent positions (Fig. 7), BI-9774 contains an endocyclic nitrogen in its core which 991 does not, and BI-9774 has a dianhydro-D-mannitol moiety attached to the C2 position of the central ring, whereas 991 has an arylcarboxylate group connected through an ether linkage at that position.

Given the lack of effect of BI-9774, it was suprising that its closely related analogue MK-8722 [15, 16, 18] partially inhibited hTASK-3 currents when applied at concentrations in excess of those required to maximally activate AMPK, given that these compounds are structurally identical bar the imino-dimethyl-lambda6-sulfanone group only present at the para position of the biphenyl ring of BI-9774 (Fig. 7). That said, over the concentration range where MK-8722 inhibits hTASK-3 (10-30 µM), it has also been shown to inhibit mitochondrial oxidative phosphorylation; briefly, Langendorf and colleagues showed that MK-8722-induced phosphorylation of α-Thr172 was diminished by ~55 % in HEK-293T/17 cells dominantly expressing the AMP-insensitive AMPK-γ1 R299G mutant, while by contrast α-Thr172 phosphorylation by other ADaM site activators remained unaffected [28]. MK-8722 may therefore attenuate hTASK-3 currents indirectly by reducing cytoplasmic ATP concentrations [30–32], which would account for its relatively slow time-course of action.

Adding to the above, we showed that hTASK-3 currents remained largely unaffected following application of 1 mM AICA-riboside, a pro-drug that is metabolised to the AMP mimetic AICA-ribotide (ZMP) and thus activates AMPK [29]. This latter finding is in accordance with previous studies on the effects of AICA-riboside on TASK currents in rat carotid body type I cells [8, 33] but runs contrary to our preliminary work, which suggested that AICA-riboside might inhibit endogenous TASK currents in rat carotid body type I cells and hTASK-3 currents stably expressed in HEK-293 cells [2, 3]. This strongly suggests that our previous contrary findings were likely due to experimenter errors, that did not take into account variations in current magnitude between control and test groups where preincubation was used [3], or current run-down in the whole-cell configuration of the patch-clamp technique, variations in seal resistance and/or changes in series resistance where acute application was used [2].

The rank order of potency for the AMPK agonists tested here adds further support to the view that A-769662 and 991 act on hTASK-3 by a mechanism independent of AMPK. The most potent inhibitor of hTASK-3 potassium currents was the AMPK-β1 selective activator 991 which exhibited very strong concentration-dependence and had an IC_50_ of 9 µM, which approximates the concentration required for maximal AMPK activation [10, 12]. By contrast, the pan-AMPK activator MK-8722 [15, 16, 18] inhibited hTASK-3 with an IC_50_ of 22 µM, which is an order of magnitude in excess of the concentration required for maximal AMPK activation [15, 17, 28]. This was followed by A-769662 which had an IC_50_ of 298 µM, once again in excess of the concentration (100 µM) required to maximally activate AMPK [19]. More strikingly still, BI-9774 had no effect on hTASK-3 potassium currents at concentrations (100 µM) an order of magnitude higher than those required to maximally activate AMPK [13, 14]. The rank order of potency by IC_50_ for inhibition of hTASK-3 by the direct AMPK activators tested here is, therefore, 991 > MK-8722 > A-769662 >>>>B-I9774, which is quite different from that for AMPK activation, which reads MK-8722 [15, 17] > BI-9774 [14] > 991 [10, 12] > A-769662 [10].

Given the above and the lack of effect on hTASK-3 of BI-9774 and AICA-riboside, the rapid and strong concentration-dependent inhibition of hTASK-3 currents by A-769662 and 991 likely represents a novel off-target effect(s) independent of AMPK activation. This was confirmed using HEK-293 cells that stably overexpressed a non-phosphorylatable AMPK-β1 S108A mutant that has been shown to block AMPK activation by compounds that bind to the ADaM site [10, 19]. Briefly, in AMPK-β1 S108A expressing HEK-293 cells we observed similar levels of hTASK-3 inhibition by A-769662 to those seen for cells expressing wild-type AMPK-β1.

Significantly, others have shown that 500 µM A-769662 does not inhibit TASK-1,TASK-3 or TASK-1/3 currents in rat carotid body type I cells [8]. Therefore, A-769662 may exhibit selectivity for hTASK-3 over rat TASK-3 and rat TASK-1/3. Supporting this view, a seach of the GenBank™ data base using the BLAST sequence alignment program [34, 35] revealed that the rat TASK-3 sequence (GeneBank accession No. Q9ES08.2) shares ~69% identity with that of hTASK-3 channels (GeneBank accession No. Q9NPC2.1). Intriguingly, the main differences are located in the C-terminus, where binding of fatty acids and other polyanionic lipids has been shown to modulate TASK-3 potassium channels [36]. It is plausible, therefore, that A-769662 and 991 might act on the same C-terminal binding site of hTASK-3 as do fatty acids and other polyanionic lipids, given that, in common with the action of A-769662 and 991, long chain fatty acyl CoAs may activate AMPK by binding to the ADaM site [37]. Interestingly in this respect, the Hill coefficient for 991, but not A-769662, indicated positive cooperativity. However, further extensive studies will be required to confirm the binding site and the mechanism of inhibition, and to determine whether or not 991 and/or A-769662 show selectivity for hTASK-3 homodimers over human TASK-1, TASK-1/3 heterodimers, TASK-5 and indeed other members of the K_2P_ potassium channel family [38].

It is notable that azabenzimidazole derivatives are also potent inhibitors of Rho kinases [39] and spleen tyrosine kinase [40], while thienopyridine derivatives are effective inhibitors of certain members of the Src family of protein kinases [41], because TASK channel currents can be reduced by both Src kinase-[42] and Rho kinase-dependent [43] signalling pathways. Such off-target effects could not explain the inhibition of hTASK-3 currents observed here. However, inhibition of Src family kinases could perhaps confer the transient increase in hTASK-3 currents elicited by A-769662 (30-500 µM). Alternatively, this transient increase in hTASK-3 current could result from inhibition of the sodium-potassium ATPase [44], and consequent transient increases in ATP availability proximal to the plasma membrane [30].

In conclusion, further investigations on the structure-activity relationships of the benzimidazole 991, the thienopyridine A-769662 and related compounds against hTASK-3 are warranted, as they will guide future drug development that could lead to new therapeutics for certain cardiovascular and neurological disorders where TASK channels have been implicated [45–47]. Importantly in this respect, 991 has not been shown to have the off-target interactions previously reported for A-769662, namely inhibition of the sodium-potassium ATPase [44] and glucose uptake [48]. Moreover, 991 and the hTASK-3-inactive 4-azabenzimidazole BI-9774 are structurally related (Fig. 7), sharing many important features including a central core. There are, however, distinguishing features in that BI-9774 contains an endocyclic nitrogen in its core which 991 does not (position 4 is CH in 991, whereas N in BI9774), and BI-9774 has a dianhydro-D-mannitol moiety attached to the C2 position of the central ring, whereas 991 has an arylcarboxylate group connected through an ether linkage at that position. We therefore have a clear and evident structure-activity relationship to work with.

## Data Availability

The datasets generated or analysed during this study available from the corresponding author upon appropriate request.
